# What evidence exists for the impact of restoration of natural processes on biodiversity in temperate ecosystems: a systematic map protocol

**DOI:** 10.1186/s13750-025-00373-6

**Published:** 2025-10-18

**Authors:** Kerstin Bouma, Pablo Villalva Aguilar, Siri Vatsø Haugum, Bjarke Madsen, Urs Albert Treier, Signe Normand, Carsten Rahbek, Jacob Heilmann-Clausen

**Affiliations:** 1 Center for Macroecology, Evolution and Climate, Globe Institute, 2100 Copenhagen Ø, Denmark; 2 Section for Ecoinformatics and Biodiversity, Department of Biology, Aarhus University, Aarhus, Denmark; 3 Center for Sustainable Landscapes Under Global Change, Department of Biology Ecoinformatics and Biodiversity, Aarhus University, Aarhus, Denmark; 4 Center for Global Mountain Biodiversity, Globe Institute, University of Copenhagen, 2100 Copenhagen Ø, Denmark; 5 Department of Biology, University of Southern Denmark, 5230 Odense, Denmark

**Keywords:** Dynamic nature management, Monitoring, Hydrology, Grazing, Fire, Open-ended restoration, Rewilding

## Abstract

**Background:**

Over the last decade, a paradigm shift has been initiated in the field of nature management and conservation with shifting the focus from traditional, more static conservation efforts to dynamic conservation efforts. To promote dynamic restoration efforts, it is essential to provide nature managers with tools to measure the impact and effectiveness of relevant interventions. However, despite increasing practice, quantifying restoration management in a relevant and measurable way remains challenging. Therefore, this systematic map aims to elucidate which metrics are being used to measure the impact of *dynamic nature management* working with natural processes.

**Methods:**

To assess which metrics are being used to measure this impact, we will perform a systematic map in Web of Science, Scopus and Agricola. In addition, we will search for grey literature through directed visits to organizational websites, search ProQuest for relevant PhD theses on the topic and perform a search in Google Scholar. For the latter, we will only consider the first 200 articles. We will include articles conducted based on research in natural areas within temperate zones, where natural dynamics (e.g., grazing, hydrology, fire) are present, introduced or restored, and are assessed using before/after or control/impact study designs. The selected studies should mention measurements of the natural process restoration outcome related to relevant biodiversity metrics (e.g., richness, diversity, abundance). Literature from review studies will be included to identify other relevant articles. All studies positively assessed as relevant through the criteria above will be subject to critical appraisal. Hereafter, we will use the critical appraisal tool as issued by Environmental Evidence. The data obtained will be used to create an overview of restoration and conservation current practices in order to identify knowledge gaps. We will disseminate our results to nature managers and provide a time- and cost- assessment of each measurement to create a guide on monitoring of dynamic nature management.

**Supplementary Information:**

The online version contains supplementary material available at 10.1186/s13750-025-00373-6.

## Background

Over the last decades, a paradigm shift has been initiated in the field of nature management [3]. The focus has moved from traditional conservation efforts, aimed at preserving certain species or habitat types, to dynamic conservation efforts, targeting the restoration of natural ecosystem processes [3]. This shift is related to concepts and practices such as rewilding, open-ended restoration and forward-looking restoration, all striving to create a foundation from which nature can naturally regenerate (self-organizing nature) [5, 12]. This self-organized nature will, in most cases, develop towards an unknown future dynamic state. There is growing knowledge on quantifying restoration efforts or determining the 'success' of a restoration project building on restored processes. However, measuring this impact in a generalizable and relevant way remains difficult [9]. To provide nature managers with information on potential impact metrics is therefore essential to support effective reporting and evaluation of conservation measures.

The framework proposed by Torres et al. [12] focused on two main pillars related to measuring rewilding progress: (1) decreasing human forcing of natural processes and (2) increasing the ecological integrity of the ecosystem. These are broad concepts that can comprise many individual metrics but are not directly translatable to field measurements. Other studies on quantifying restoration efforts have focused on measurements of trophic complexity [4, 8], landscape metrics or ecosystem structure [4, 10, 13], and ecological or biophysical processes [5, 6, 8]. Yet, the indicators that could be measured in the field to validate and quantify these concepts and restoration success are not necessarily clear from their definitions.

Downscaling these broad concepts to measurable metrics is challenging if the aim is to capture dynamics at site level during restoration. Yet, such metrics are vital to promoting evidence-based nature management. This review aims to research which metrics have been and are currently being used, and which of these would be suitable for quantification of dynamic restoration. Specifically, we will focus on methods that go beyond the site scale and can be used on a landscape scale (i.e. are scalable). Furthermore, we will include fast technological developments such as LiDAR, satellite imagery, eDNA, and automatic image detection. In the context of this review, we will use the term dynamic nature management to describe interventions that focus on restoration of natural processes and interventions defined as open-ended restoration, forward-looking restoration, or rewilding.

### Stakeholder engagement

As an outcome, this study aims to provide an overview of useful metrics and set the bases for creating a guide for nature managers working with restoration of ecological processes in temperate zones. The original idea for this review emerged from close collaboration with nature managers in Denmark, specifically with Aage v. Jensen Naturfund, who manage multiple areas in Denmark covering a wide range of habitat and size. They highlighted a knowledge gap in monitoring, guiding and reporting practices of dynamic nature management, specifically, approaches that support goal setting and progress tracking while accommodating the inherent dynamics and stochasticity of nature. An overview of which metrics are often used when assessing restoration efforts and their cost- and time effectiveness in monitoring programs is needed to guide quantification of dynamic restoration.

## Objective of the systematic map

The purpose of this systematic map is to elucidate key metrics that can be used to quantify the impact of dynamic nature management working with restoration of natural processes. We aim to provide a guide on how to measure restoration progress in terrestrial and aquatic-terrestrial gradients in temperate nature areas. We will focus on impacts due to restoration of processes related to herbivory, water level dynamics, fire and forest gap dynamics.

### Primary question

What evidence exists for the impact of restoration of natural processes on biodiversity in temperate ecosystems?

Hence, we will investigate the metrics that are being used to quantify the impact of restoration of natural processes on biodiversity in temperate ecosystems.

Components of the primary question that are used to structure our search terms and delineate our eligibility criteria:

**Table:** 

Population	Intervention	Comparator	Outcome
Nature areas undergoing dynamic nature management OR undergoing spontaneous development due to natural processes in temperate climate zones	Restoring natural dynamics/processes OR spontaneous development within natural areas	No restoration of natural dynamics/processes OR comparison to measures from before restoration of natural dynamics/processes OR time series of spontaneous nature development	Biodiversity metrics (e.g., richness, diversity, abundance, landscape metrics (spatial), functional traits)

## Methods

The systematic review will follow the Collaboration for Environmental Evidence Guidelines [1] and Standards for Evidence Synthesis in Environmental Management and will conform to the ROSES reporting standard for systematic maps [2] (see Appendix 1).

### Searching for articles

Our search string (Table 1) searches for literature including open-ended restoration or rewilding concepts (type of restoration). We focus on research on dynamic nature, stochastic disturbances or natural development (type of development). By including these terms in the search string, we will filter out the articles focusing on other types of restoration approaches. We are interested in finding articles looking into the impact on ecosystem development across all taxonomic groups.

**Table 1:** Overview of the search terms in relation to different topics of the systematic map

Type of restoration	Type of development	Biodiversity	Ecological processes
Rewild* OR restor*	dynamic* OR disturbance* OR “natural development*” OR “spontaneous development*” OR “natural process*” OR “natural colonization” OR “natural colonisation” OR “spontaneous colonization” OR “spontaneous colonisation” OR “autogenic colonization” OR “autogenic colonisation” OR “natural succession” OR “spontaneous succession” OR “autogenic succession”	abundanc* OR diversity OR richness OR function*	Graz* OR brows* OR wallow* OR root* OR tramp* OR “water level dynamic*” OR “water level fluctuation*” OR “water level drawdown” OR hydrodynamic* OR “flooding dynamic*” OR fire OR “gap cutting” OR “partial cutting” OR “canopy gap*”

The search in the bibliographic databases will be conducted in English. For the organizational websites and web-based search engines (grey literature) we will investigate English, Spanish, Danish, Norwegian, Swedish, Dutch and German literature.

The main search will be done in Web of Science (Core collection, all indices, access through University of Copenhagen, search in topics (TS)), Scopus (Search in title, abstract, keywords) and Agricola (through the Ovid interface, search in keyword). We refined the search to “article” or “review article”, this yielded a total of 3.852 studies in Web of Science and 3.032 studies in Scopus and 1306 in Agricola (3-7-2025). After removal of duplicates this resulted in 5232 articles in total. We will also search ProQuest for relevant PhD thesis on the topic and google scholar (using Harzing’s Publish or Perish). For the latter, we will only consider the first 200 articles, using a shorter search string to fit the character limit ((rewild* OR restor*) AND (abundanc* OR diversity OR richness OR function*) AND (Graz* OR brows* OR wallow* OR root* OR tramp* OR “water level” OR fire OR “gap cutting”) AND (dynamic* OR disturbanc* OR natural)). Review articles will only be used to assess new relevant articles and will not be included in the systematic map itself.

Additionally, we will search reports on organizational websites:

- Ontwikkeling en Beheer Natuurkennis (OBN, www.natuurkennis.nl) (Dutch NGO)
- Staatsbosbeheer (www.staatsbosbeheer.nl) (Governmental nature manager)
- Natuurmonumenten (www.natuurmonumenten.nl) (Dutch NGO)
- ARK Rewilding (www.arkrewilding.nl) (Dutch NGO)
- Rewilding Europe (www.rewildingeurope.com)
- Aage v. Jensen naturfund (https://www.avjf.dk/avjnf/) (Danish nature organisation)
- Saksfjed Wilderness (www.vildmarken.dk) (Danish nature organisation)
- Naturstyrelsen (https://naturstyrelsen.dk/) (Governmental nature manager)
- DCE (DCE—Nationalt Center for Miljø og Energi)
- Bora (Bergen Open Research Archive, https://www.bora.uib.no) (University of Bergen (Norway), covering PhD thesis, MSc thesis, reports and other academic publications)
- Norwegian Institute of Bioeconomics (Norwegian Research Institute, https://www.NIBIO.no/publications)
- Norwegian Institute of Nature Research (Norwegian Research Organisation, https://www.nina.no/Om-NINA/Publikasjoner)
- Norwegian Environment Agency (miljodirektoratet.no)
- NTNU Open (Norwegian University of Science and Technology, ntnuopen.ntnu.no)
- UiO DUO (University of Oslo (Norway), duo.uio.no)
- Norwegian University of Life Sciences (https://brage.nmbu.no)
- UiT Munin (University of Tromsø (Norway), munin.uit.no)
- SLU (Swedish University of Agricultural Sciences, https://pub.epsilon.slu.se/)
- Swedish Environmental Protection Agency (https://www.naturvardsverket.se/publikationer/)
- Forestry Research Institute of Sweden (www.skogforsk.se)
- Consejo superior de investigaciones científicas (https://www.csic.es) including the repository (https://digital.csic.es)
- Estación biological de Doñana (https://www.ebd.csic.es/en/about-us/annual-reports)
- Ministerio de transición ecológica y reto demográfico (https://www.miteco.gob.es/es.html)
- Organismo autónomo Parques Nacionales (https://www.miteco.gob.es/es/red-parques-nacionales/)
- Centre de Recerca Ecological i aplicacions forestals (https://www.creaf.cat)
- EU-LIFE projects (Explore Natura 2000)

### Comprehensiveness of the search

To determine the search precision, we have checked the presence of a list of benchmark articles in our results (Appendix 2). After doing a preliminary search in Web of Science, Scopus, Agricola and Google Scholar, we checked the comprehensiveness of the search and found all our benchmark articles back.

### Search update

We will update the search every year on the first of January that the protocol is not submitted.

## Article screening and study eligibility criteria

### Screening process

COVIDENCE will be used as a tool to facilitate the screening strategy. Duplicates will be removed. All remaining articles will be screened by reviewing their title and abstracts based on the eligibility criteria. In case of uncertainty, the reviewer will tend towards inclusion at this stage. The articles passing these criteria will be assessed for inclusion based on the full text screening. Reviewers will not screen articles authored by themselves. After screening 200 articles, a meeting will be held with the reviewers to assess exceptions and ambiguous cases and adjust the inclusion criteria if needed.

Before starting the review, 50 papers will be randomly selected and reviewed by all reviewers (± 6) independently to check for consistency. After this, reviewers will meet to discuss and compare results to be able to uncover discrepancies in inclusion/exclusion criteria and further sharpen them. Discrepancies during this pilot will be measured and reported using Cohen’s Kappa values. Cohen’s Kappa values > 0.6 will be considered an accepted level of agreement. The title/abstract and full-text screening will be done by two reviewers independently. If studies are categorized as unclear (indicated by a discrepancy of the two reviewers) during the title/abstract screening, the article will be passed to full-text screening, to enable decision making based on all the available information. If there is a disagreement during the full-text screening, the two reviewers will meet and discuss their line of reasoning and try to reach a consensus. If no consensus can be reached, a meeting with a third reviewer will be held. The decision of the third reviewer will then be decisive (majority vote).

### Eligibility criteria

*Relevant subjects*: Nature areas in the temperate zone.

Here, we focus on terrestrial ecosystems and aquatic-terrestrial gradients including, but not exclusively, forests, grasslands, heathlands and dunes. Studies on wetlands, peatlands, coastal areas and riparian habitat will also be considered if the study is not conducted solely in the aquatic environment. Temperate areas are identified following the Köppen-Geiger climate zones. Boreal and Mediterranean areas will be considered for review as there is a potentially large amount of literature on restoration projects under relatively similar climatic conditions. Tropical, polar, desert or alpine areas will be excluded from review to make this review operable and generalizable. (Table 2).

**Table 2:** Overview of inclusion and exclusion criteria during study screening

Criterion	Include	Exclude
Climate zone	Temperate	Tropical, Polar, Desert, Apine
Ecosystem Type	Terrestrial, semi-terrestrial transition zones	Marine, aquatic-only
Restoration focus OR natural ecosystems	Dynamic, forward-looking, process-based or natural processes	Static species/habitat restoration without process focus or without natural processes
Metrics Reported	Field-based biodiversity/process metrics (quantitative)	No metrics mentioned or only conceptual frameworks
Study Type	Empirical field studies, BACI, impact studies, literature reviews	Modelling-only, purely genetic focus
Language	English (primary), Dutch, Spanish, German, Swedish, Norwegian, Danish	Other languages

*Relevant type of intervention*: Restoration or introduction of natural dynamics.

In this review we will focus on several natural dynamics. 1. Grazing or browsing as a strategy to restore vegetation heterogeneity. This can be assessed by observing the impact of natural grazing regimes or by manipulating herbivores densities, such as fencing, introducing or culling of animals. 2. Hydrology and water level dynamics. This can include studies that focus on physical adaptations such as dam removal, closure of drainage pipes or ditches, reconnecting areas, on manually changing the water level or inducing a drawdown or the impact of natural water level dynamics. 3. Fire. This can be natural fires or prescribed fires done on a regular basis. These factors will be evaluated in the context of other co-occurring ecosystem processes, not least succession (Table 2).

*Relevant type of comparator*: Non-intervention or before intervention.

Both temporal and spatial comparisons for the implementation or observation of natural dynamics will be considered. Here, we can make use of both the before/after (BA) measurements and control/impact (CI) measurements or a combination (before/after/control/impact; BACI design) (Table 2).

*Relevant type of outcome*: Metrics used to measure the outcome of the restoration intervention or the impact of natural development.

This can be a wide range of metrics on various groups of organisms. It can focus on, but is not limited to, abundance, diversity, composition, trophic complexity, functional guilds, species traits, structural indices, woody regeneration (seedlings/saplings), seed dispersal, ecosystem heterogeneity, landscape metrics. All organism groups will be considered in the review. Also, the impact on abiotic ecosystem processes like fire or infiltration will be considered. We will group the metrics by four widely used types: spatial metrics, functional metrics, structural metrics and metrics on species composition [11] (Table 2).

*Relevant type of study*: Field studies, review studies and (non-scientific) reports.

We are primarily interested in field studies. Review studies on specific ecosystem types or processes will be included to find new relevant articles. We will also include non-scientific reports in our review. We will exclude modelling studies, policy discussions and perspective papers (Table 2).

*Language*: Full text written in English. This will be broadened to Danish, Dutch, Spanish, Swedish, Norwegian and German for the grey literature (Table 2).

*Reasons for exclusion*: We will provide a list of articles excluded at the full text stage with reasons for exclusion.

## Study validity assessment

Studies that have been assessed as relevant through the criteria above will be subject to critical appraisal. Therefore, we will use the critical appraisal tool as issued by Environmental Evidence [7]. This tool is designed for assessing risk of bias in primary research studies. The tool provides a structured and transparent way of evaluating the quality and relevance of environmental conservation evidence. Based on this assessment, studies will be categorized as high, medium or low susceptibility to bias in their outcome. Studies highly susceptible to bias will be excluded from review. The critical appraisal will be done by two reviewers. In case of doubtful cases, a meeting is held to reach a consensus. If this cannot be achieved, a third person will be asked to aid in the final decision, and a majority vote will be cast.

## Data coding strategy

The focal data for this revision is merely qualitative and focuses on the metrics used in following restoration of natural dynamics in a study area. We will extract the following metadata for each article (Appendix 3):

- Monitoring period (start date; end date; frequency of sampling; time since restoration/monitoring)
- Taxa that are monitored (vegetation, birds, insects, fish, invertebrates, reptiles, amphibians, fungi, micro-organisms, etc.)
  - We will create one row per taxa measured
- Type(s) of ecosystem (wetland, forest, heathland, grassland etc.)
- Design (before-after, control-impact, or BACI)
- Type of implemented actions for restoration (if not focused on natural processes)
- Ecological processes involved (fire, grazing, browsing, hydrology, water level dynamics, forest gap dynamics etc.)
- Metrics to measure the impact of the natural ecological process or the impact of the intervention measure (this can also be restoring or introducing the natural process).
- Type of metric (spatial, temporal, species composition, structural, functional)
- Direction of the natural ecological process or restoration intervention (positive, neutral or negative)
- Geographical coordinates (geographical center of the study area; multiple locations possible, in degrees)
- Type and size/length of plots that are being measured (polygons, transects, points, ha, m)
- Scale of study (local (one area), regional (multiple areas), national, global)
- Number of plots within area
- History of land use (baseline, history of management/restoration)

If information is not available, we will try to contact the authors to obtain complete information. If the geographical coordinates are not given, these will be extracted based on the study site name mentioned in the article.

## Study mapping and presentation

One of the main goals is to gain insights into knowledge gaps in monitoring ecological processes in relation to dynamic nature management. By aggregating the known information in tables and figures and through discussions with nature managers, we will identify key knowledge gaps and knowledge clusters in this field.

The studies selected for the review and their results will be described in a narrative synthesis, i.e., a qualitative description of the studies and their results. We will provide an overview of the steps of the review process based on the ROSES flow diagram [2]. We will provide figures from the collected data on for example the geographic distribution of the included studies, the distribution of studies across ecosystem types and the type of ecological processes that are being measured. We will group articles based on metrics on similar ecological processes and give an overview of which metrics can be used to monitor each of them and for which kind of organisms. For dissemination purposes, we want to create an overview of the cost- and time- effectiveness of each metric. Furthermore, we will describe the available evidence, highlight knowledge gaps and will generally evaluate the existing body of evidence. To follow procedural independence, any study authored by one of the systematic reviewers will be assessed by other reviewers at every stage of the process.

## Supplementary Information

Supplementary material 1.

Supplementary material 2 .

Supplementary material 3.

## Data Availability

No datasets were generated or analysed during the current study.
