# Supplementary material for: What evidence exists for the impact of restoration of natural processes on biodiversity in temperate ecosystems: a systematic map protocol
Source: Environ Evid. 2025 Oct 18;14:18. doi: 10.1186/s13750-025-00373-6 (PMC12535033; doi:10.1186/s13750-025-00373-6)
Supplement: Supplementary file 2 — Supplementary material 2 . [file 13750_2025_373_MOESM2_ESM.pdf]

README – Appendix II

Benchmark articles for comprehensiveness of search

Here we provide a list of eleven articles that were selected beforehand to test the comprehensiveness of the search. All articles were retrieved based on our current search string.

Appendix II

Benchmark articles for comprehensiveness of search

1. Alderson, Rachel, Casper HA van Leeuwen, Elisabeth S. Bakker, Kerstin Bouma, Han Olff, Valérie C. Reijers, Stefan TJ Weideveld et al. "Active wetland restoration kickstarts vegetation establishment, but natural development promotes greater plant diversity." *Journal of Applied Ecology* 62, no. 5 (2025): 1166-1176.
2. Ejrnæs, D.D., Olivier, B., Bakker, E.S., Cornelissen, P., Ejrnæs, R., Smit, C. et al. (2024) Vegetation dynamics following three decades of trophic rewilding in the mesic grasslands of Oostvaardersplassen. *Applied Vegetation Science*, 27, e12805. Available from: https://doi.org/10.1111/avsc.12805
3. Kerns, B. K., M. Buonopane, W. G. Thies, and C. Niwa. 2011. Reintroducing fire into a ponderosa pine forest with and without cattle grazing: understory vegetation response. Ecosphere 2(5):art59. doi:10.1890/ES10-00183.1
4. Gottlieb, L., Schäfer, B. A., & Buttenschøn, R. M. (2024). European bison (Bison bonasus) increase plant species richness in forest habitats. *Forest Ecology and Management*, *561*, 121891.
5. Pinto-Cruz, C., Matono, P., Almeida, E., Meireles, C., Fernandes, M. P., Ferreira, L., & Belo, A. D. (2023). How can Mediterranean temporary ponds benefit from disturbance? Challenges and lessons learned from vegetation management. *Restoration Ecology*, *31*(8), e14045.

6. Żmihorski, M., Pärt, T., Gustafson, T., & Berg, Å. (2016). Effects of water level and grassland management on alpha and beta diversity of birds in restored wetlands. *Journal of applied ecology*, *53*(2), 587-595.
7. Burrascano, S., Copiz, R., Del Vico, E., Fagiani, S., Giarrizzo, E., Mei, M., Mortelliti, A., Sabatini, F.M. and Blasi, C., 2015. Wild boar rooting intensity determines shifts in understorey composition and functional traits. *Community Ecology*, *16*(2), pp.244-253.
8. Geest, G. V., Wolters, H., Roozen, F. C. J. M., Coops, H., Roijackers, R. M. M., Buijse, A. D., & Scheffer, M. (2005). Water-level fluctuations affect macrophyte richness in floodplain lakes. *Hydrobiologia*, *539*(1), 239-248.
9. Kärvemo, Simon, Christer Björkman, Therese Johansson, Jan Weslien, and Joakim Hjältén. "Forest restoration as a double-edged sword: The conflict between biodiversity conservation and pest control." *Journal of Applied Ecology* 54, no. 6 (2017): 1658-1668.
10. Shackelford, N., Renton, M., Perring, M. P., Brooks, K., & Hobbs, R. J. (2015). Biodiversity change in heathland and its relationships with shifting local fire regimes and native species expansion. *Journal of Plant Ecology*, *8*(1), 17-29.
11. Smit, C., Ruifrok, J. L., van Klink, R., & Olff, H. (2015). Rewilding with large herbivores: The importance of grazing refuges for sapling establishment and wood-pasture formation. *Biological Conservation*, *182*, 134-142.
