# Supplementary material for: What evidence exists for the impact of restoration of natural processes on biodiversity in temperate ecosystems: a systematic map protocol
Source: Environ Evid. 2025 Oct 18;14:18. doi: 10.1186/s13750-025-00373-6 (PMC12535033; doi:10.1186/s13750-025-00373-6)
Supplement: Supplementary file 3 — Supplementary material 3. [file 13750_2025_373_MOESM3_ESM.pdf]

README – Appendix III

Overview of metadata collection

ID – Number of entries, each combination of article and measured taxa is one row

Author – Authors of the paper included in the review

Year – Year in which the paper was published

Journal – Journal in which the paper was published

Coordinates – Coordinates of the location of the study site. If more than one site is used the coordinates for all the sites will be extracted if possible (unit = degrees).

Monitoring period – Start date (at least year) and end date (at least year), also the covered monitoring period will be shown. (unit start and end date: ddmmyyyy; unit period: days, months or years).

Taxa – Which group of organisms is being measured (e.g. vascular plants, bryophytes, epiphytes, trees, birds, amphibians, reptiles, mammals, insects, fish, fungi).

Ecosystem type – The type of ecosystem that is being measured (e.g. wetland, forest, heathland, grassland), can be multiple.

BACI – How is the data collected. Before-after intervention (BA), control-impact of intervention (CI) or a combination of both (BACI).

Ecological processes – Which ecological processes are studied in the paper. (e.g. fire, grazing, water level dynamics, forest gap dynamics)

Indicators – Which metrics are being used to measure the impact of the restoration of the ecological process in the study area

Type of metric – Classify the different indicators into broader groups such as spatial metrics, temporal metrics, species composition, structural metrics or functional metrics.

Size of plots – Type and size of the plots that were studied (unit: ha)

Scale of study – local (one area), regional (multiple areas), national, global

Number of locations – Number of plots within an area

Land use history – Short description of the history or management of the area.

Restoration effort – Which type of actions for restoration (if not focused on natural processes) are being implemented.

Direction of restoration – What is the impact of the restoration of natural processes on the measured taxonomic groups according to the measured metrics (positive, negative, neutral).

APPENDIX III Overview of metadata collection

ID	Author	Year	Journal	Coordinates	Monitoring period	Taxa	Ecosystem type	BACI	Ecological processes	Indicators	Type of metric	Size of plots	Scale of study	Number of locations	Land use history	Restoration effort	Direction of restoration
1	Gottlieb et al.	2024	Forest Ecology & Management	55°11´N, 14°94´E	2012-2020 (8 years)	Vascular plants	Forest	CI	Grazing	Species richness, abundance	Species composition	Radius of 5m (i.e. 78.5m2)	Local	50 enclosure plots (10 per five most dominant forest types), 30 control plots	Unknown	Species reintroduction	Positive
2	Gottlieb et al.	2024	Forest Ecology & Management	55°11´N, 14°94´E	2012-2020 (8 years)	Bryophytes	Forest	CI	Grazing	Species richness, abundance	Species composition	Radius of 5m (i.e. 78.5m2)	Local	50 enclosure plots (10 per five most dominant forest types), 30 control plots	Unknown	Species reintroduction	Positive
3	Kerns et al.	2011	Ecosphere	44°06'04.9"N 118°48'49.7"W	2002-2007 (6 years)	Vegetation	Forest	BACI	Fire, grazing	Woody fuels, litter and duff accumulation, woody debris, plant cover, density, and height, richness, and reproductive capability (flowering stem height and density), understory plant canopy cover, Ground cover (e.g., bare soil, rock, litter, woody debris >10 cm diameter), maximum grass leaf height, maximum flower stem height, and number of flowering stems for each grass species, species presence, shrub cover, conifer regeneration, overstory tree canopy cover. Species richness and community composition.	Structural metrics, species composition	10 m radius subplots (within burning treatment) for grazing. (Each subplot has eight 1-m2 quadrats for some vegetation monitoring)	Local	4 blocks based on stand with three units for the burning treatment (control, spring 5year interval, fall 5year interval). With Per unit (12) there are three grazed and three ungrazed plots (n=72)	Thinning was done in 1994, and burning also occurred in 1997 -1998 and is now being reburned (2002-2003)	Thinning, reburning and grazing	Grazing: neutral; Fire: Positive
4	Zmihorski et al.	2015	Journal of Applied Ecology	5 sites in Sweden	4 years	Birds	Wet grasslands	CI	Grazing, hydrology	Species richness, presence, diversity, species turnover (beta diversity)	Species composition	3.1 ha	National	137 sites within 5 wet grasslands	Long management history (i.e. several hundred years) for fodder production (hay cutting) and cattle grazing, without fertilization or cultivation.	Water level management, vegetation removal and grazing	Grazing and flooding: positive
5	Zmihorski et al.	2015	Journal of Applied Ecology	5 sites in Sweden	4 years	Vegetation	Wet grasslands	CI	Grazing, hydrology	Proportion of open forest and proportion of open water	Spatial metrics	3.1 ha	National	137 sites within 5 wet grasslands	Long management history (i.e. several hundred years) for fodder production (hay cutting) and cattle grazing, without fertilization or cultivation.	Water level management, vegetation removal and grazing	Grazing and flooding: positive
